# A comprehensive review on rice responses and tolerance to salt stress

**DOI:** 10.3389/fpls.2025.1561280

**Published:** 2025-03-31

**Authors:** Obed Kweku Sackey, Naijie Feng, Yushawu Zakaria Mohammed, Chrystella Fernanda Dzou, Dianfeng Zheng, Liming Zhao, Xuefeng Shen

**Affiliations:** ^1^ College of Coastal Agricultural Sciences, Guangdong Ocean University, Zhanjiang, China; ^2^ National Saline-Tolerant Rice Technology Innovation South China Center, South China, Zhanjiang, China; ^3^ College of Applied Economics, Guangdong Ocean University, Zhanjiang, China

**Keywords:** salt tolerance, spermidine treatments, quantitative trait loci (QTLs), global food security, CRISPR-Cas9 applications, oryza sativa

## Abstract

The challenge of salinity stress significantly impacts global rice production, especially in coastal and arid regions where the salinization of agricultural soils is on the rise. This review explores the complex physiological, biochemical, and genetic mechanisms contributing to salinity tolerance in rice (*Oryza sativa* L.) while examining agronomic and multidisciplinary strategies to bolster resilience. Essential adaptations encompass the regulation of ionic balance, the management of antioxidants, and the adjustments to osmotic pressure, all driven by genes such as OsHKT1;5 and transcription factors like OsbZIP73. The evolution of breeding strategies, encompassing traditional methods and cutting-edge innovations, has produced remarkable salt-tolerant varieties such as FL478 and BRRI dhan47. The advancements in this field are enhanced by agronomic innovations, including integrated soil management, crop rotation, and chemical treatments like spermidine, which bolster stress tolerance through antioxidant activity and transcriptional regulation mechanisms. Case studies from South Asia, Sub-Saharan Africa, the Middle East and, Australia demonstrate the transformative potential of utilizing salt-tolerant rice varieties; however, challenges persist, such as the polygenic nature of salinity tolerance, environmental variability, and socioeconomic barriers. The review highlights the importance of collaborative efforts across various disciplines, merging genomic technologies, sophisticated phenotyping, and inclusive breeding practices to foster climate-resilient and sustainable rice cultivation. This work seeks to navigate the complexities of salinity stress and its implications for global food security, employing inventive and cohesive strategies to confront the challenges posed by climate change.

## 1 Introduction

Rice (*Oryza sativa* L.) is a cornerstone of global food security, acting as the essential dietary staple for more than half of the world’s populace, particularly in regions such as Asia, Africa, and Latin America. This crop plays a crucial role in the caloric and protein consumption of billions, as nations in Asia account for more than 90% of the worldwide rice production and consumption (Dutta et al., 2019). The versatility of rice in thriving under various environmental conditions has facilitated its growth in a range of ecosystems, spanning from lowland paddies to upland rain-fed systems (Neupane et al., 2020; Gairola et al., 2024). Beyond its nutritional significance, rice cultivation serves as a foundation for the livelihoods of countless smallholder farmers, especially in developing nations where it represents an essential source of income and employment (Asma et al., 2023; Singh et al., 2024). The influence of the crop permeates various dimensions, shaping cultural practices, traditions, and the economic landscapes of numerous nations. In areas such as Sub-Saharan Africa, the growth of rice farming responds to the interrelated issues of food security and rural development (Saito et al., 2023). However, addressing the increasing demand for rice presents significant challenges. The surge in population and the expansion of urban areas are propelling a significant escalation in the necessity for rice production, anticipated to increase by 25% by the year 2030 to satisfy worldwide demand. The sustainability of rice production systems is further jeopardized by environmental challenges, including salinity, water scarcity, and the impacts of climate change (Ansari et al., 2023). In light of these challenges, advancements in breeding, biotechnology, and crop management, exemplified by the creation of climate-resilient rice varieties, provide a promising avenue for maintaining production levels and safeguarding global food security.

**Figure 1:**
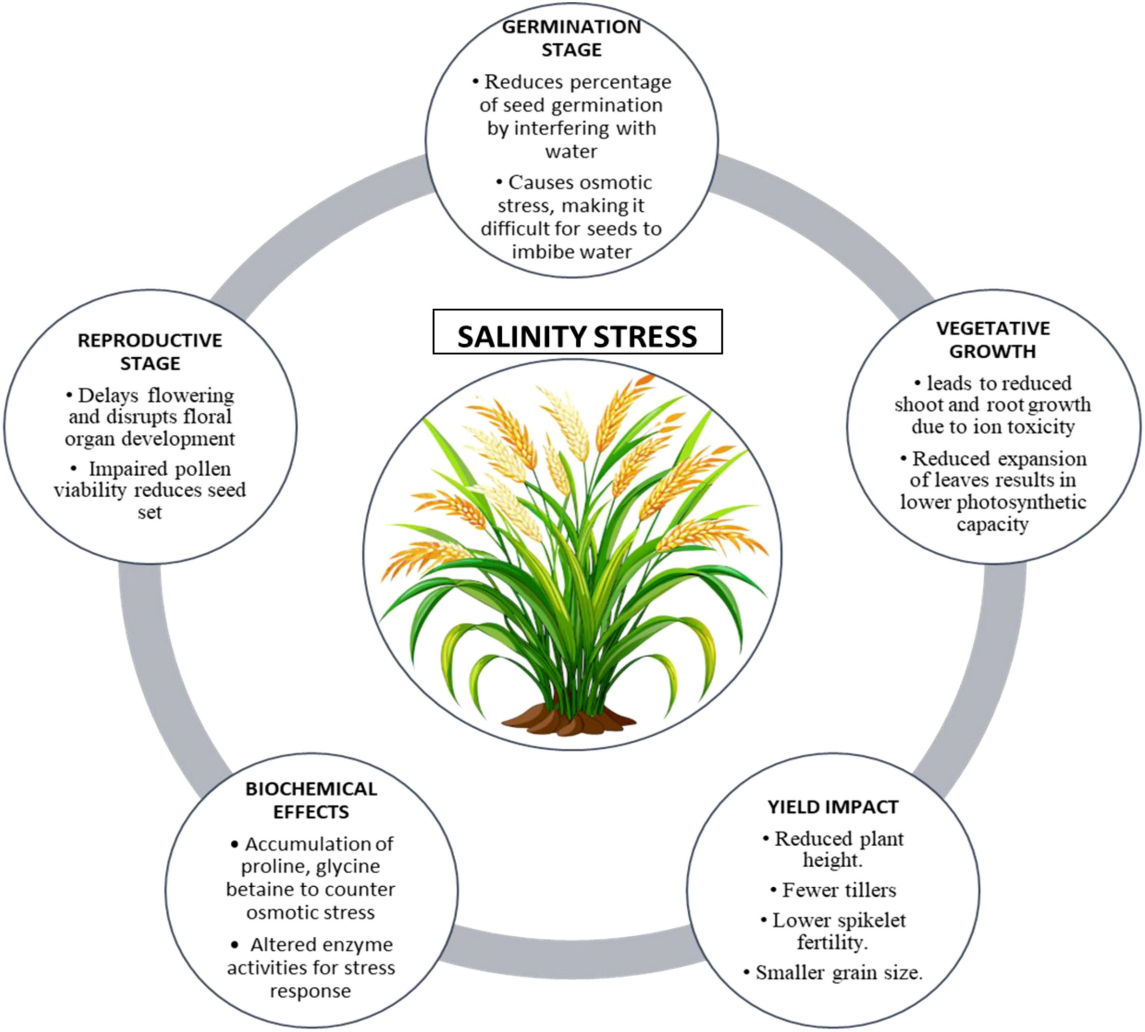
Illustrative summary of salinity stress on rice growth, development, and yield.

Salinity stress is a major abiotic challenge that profoundly influences rice production worldwide, especially in coastal and irrigated lowland regions where soil salinization is widespread. Approximately 20% of the world’s irrigated land experiences the detrimental effects of salinity, which leads to a decrease in rice yield as a result of ionic toxicity, osmotic stress, nutrient imbalance, and oxidative damage, as illustrated in **Figure 1**. *O*. *sativa* L. demonstrates a significant vulnerability to salinity, leading to pronounced growth inhibition and a decline in productivity when subjected to salt-affected soils, particularly during its early developmental phases (Abbas et al., 2024). Salinity causes an excessive buildup of sodium (Na^+^) and chloride (Cl^−^) ions in plant tissues, which disrupts ionic homeostasis and reduces the absorption of vital nutrients such as potassium (K^+^) and calcium (Ca²^+^). The ionic imbalance disrupts enzymatic activities, cellular functions, and the process of photosynthesis. Osmotic stress resulting from salinity diminishes water availability, reducing cell turgor and inhibiting growth. Excessive reactive oxygen species (ROS) lead to oxidative stress, which results in additional damage to cellular structures (Gupta et al., 2024; Požgajová et al., 2024; Ruksaar, 2024).

Recent advancements in breeding, including the incorporation of salt-tolerant genes and quantitative trait loci (QTLs) such as Saltol, have facilitated the creation of resilient rice varieties, exemplified by FL478. These varieties demonstrate improved ionic regulation and osmotic adjustment. Molecular tools, including CRISPR/Cas9, are being utilized to target and enhance stress-responsive genes, such as OsHKT1;5 and OsbHLH024, to improve salinity tolerance (Akhlasur Rahman et al., 2024). Salinity stress is expected to intensify due to climate change and the rising scarcity of water resources. Implementing effective strategies such as enhanced irrigation practices, soil management, and genetic improvement is crucial for sustaining rice production in saline-prone regions (Irin et al., 2024; Sikder and Khan, 2024). Integrating traditional breeding methods with contemporary biotechnological techniques is essential for maintaining productivity and tackling global food security issues (Zhang et al., 2024a).

This review aims to thoroughly examine the effects of salinity stress on rice (*Oryza sativa L.)* production, emphasizing the physiological, biochemical, and molecular pathways that contribute to salt tolerance in rice. It explores advancements in breeding strategies, genetic modifications, and agronomic interventions designed to mitigate the detrimental effects of high salinity and sustain yield stability in affected regions. Given the escalating challenges posed by climate change, irrigation practices, and soil deterioration, this study also assesses the global implications of salt-induced stress and evaluates region-specific adaptation strategies, particularly in vulnerable agricultural areas.

## 2 Environmental factors influencing salt stress tolerance in rice

### 2.1 Role of soil microbiome in salinity tolerance

The soil microbiome is essential for plant growth and resistance, especially under abiotic stressors such as salinity (Chen et al., 2024; Etesami, 2024). Soil microbes, such as rhizobacteria, mycorrhizal fungi, and other beneficial microorganisms, interact with plant roots and affect essential physiological processes that alleviate the impacts of salinity stress (El-Aal et al., 2024; Yahyaoui et al., 2024; Zhang et al., 2024b). Microbial communities are widely acknowledged for their capacity to improve plant resilience to diverse stresses, such as drought, nitrogen shortage, and salinity, via numerous pathways (Ali et al., 2024; Muhammad et al., 2024).

#### 2.1.1 Beneficial soil microbes in salinity tolerance

Rhizobacteria and mycorrhizal fungi are two of the many types of soil microbes that play a crucial role in improving plant health and resilience to stress. Plants benefit from rhizobacteria, which reside in close proximity to their roots, since these microbes increase nutrient intake, produce chemicals that stimulate plant growth, and cause a defense mechanism against diseases (Chaudhary et al., 2024; Hiremath et al., 2024). Improving water intake or changing the ionic balance within plant tissues are two ways in which some rhizobacteria might reduce salinity stress (Basu et al., 2023; Acharya et al., 2024). For instance, Bacillus and Pseudomonas species enhance salt tolerance by producing exopolysaccharides, which help maintain cell turgidity under saline conditions (N et al., 2024). Similarly, mycorrhizal fungi, which create symbiotic associations with plant roots, are essential for alleviating salinity stress. These fungi enhance the root system via their hyphal networks, improving water and nutrient absorption, particularly in saline soils where nutrient availability is frequently restricted. Recent research indicate that mycorrhizal inoculation can markedly enhance the salt tolerance of crops like wheat and rice by improving the plant’s capacity to sustain osmotic equilibrium and mitigate ion toxicity (Basit et al., 2024; Chaudhary et al., 2024). Mycorrhizal fungi can boost the synthesis of stress hormones such as abscisic acid, enhancing the plant’s response to salt stress (Aizaz et al., 2024).

#### 2.1.2 Microbial-mediated stress alleviation mechanisms

Soil microbes assist plants in managing salt stress via many methods, such as osmotic adjustment, ion homeostasis, and the control of plant hormones. Certain beneficial microorganisms synthesize suitable solutes (e.g., trehalose, proline) that assist plants in conserving water and sustaining cellular function in saline environments (Hmidi et al., 2018; Shafi et al., 2019; Imhoff et al., 2020; Gil et al., 2023). These solutes can concentrate in plant cells, preserving turgor pressure and stabilizing proteins and membranes, which is essential for survival in saline soils. Additionally, microbial communities play a key role in ion homeostasis by the regulation of sodium (Na^+^) and potassium (K^+^) ions in plant tissues, inhibiting the harmful buildup of Na^+^ and enhancing the absorption of K^+^ (Liu et al., 2023; Dong et al., 2024; Dreyer et al., 2024). Rhizobacteria can modulate the expression of salt-excluding or salt-tolerant genes in plants, hence assisting in the maintenance of ion equilibrium in the root zone (Guo et al., 2024). Specific bacterial strains, including Bacillus subtilis, have demonstrated the ability to augment the function of Na^+^/H^+^ antiporters in plant roots, hence mitigating salt absorption (Pabuayon et al., 2021; Song et al., 2023). Furthermore, beneficial microorganisms can stimulate the expression of plant stress-responsive genes, enhancing the plant’s capacity to endure oxidative damage caused by salt.

#### 2.1.3 Recent findings on plant-microbe interactions

Recent research efforts provide insight into the intricate relationships between plants and their corresponding soil microbiomes under saline stress. A significant discovery is the influence of plant-associated microbiomes on the plant’s transcriptional response to salinity. Research indicate that beneficial bacteria can modify the expression of essential genes associated with stress signaling pathways, including those linked to abscisic acid (ABA) production, osmotic control, and ion transport (Davis et al., 2024; Wang et al., 2025). For instance, Salt-tolerant rhizobacteria, such as *Microbacterium ginsengiterrae* S4, enhance rice resilience by increasing antioxidant enzyme activity and regulating osmotic substances under saline stress (Ji et al., 2024).

Another notable discovery is the influence of microbial metabolites on enhancing plant health. Certain microbial species generate volatile organic compounds (VOCs) that improve plant salinity tolerance by regulating the plant’s hormonal equilibrium, namely by elevating auxin and cytokinin levels, which facilitate root development and salt resistance (Barghi and Jung, 2023; Mahadik and Kumudini, 2024). Current research on plant-microbe interactions underscores the potential for microbiome-based strategies to enhance conventional breeding methods in the creation of salinity-tolerant crops. Microbial inoculants, comprising consortia of beneficial bacteria and fungi, are being studied as economical and sustainable solutions for improving crop performance in salt conditions. Microbiome-based techniques may be incorporated into precision agriculture operations, facilitating more effective management of salt-affected soils. The soil microbiome’s function in facilitating salinity tolerance is an increasing area with significant potential for sustainable agriculture. Further studies into plant-microbe interactions and their utilization in saline agriculture may substantially improve crop resilience to salinity, hence aiding food security in salinity-affected areas.

### 2.2 Combined abiotic and biotic stresses

The interaction between abiotic and biotic stressors in plants is a difficult challenge that profoundly affects worldwide agricultural growth and yield. Abiotic factors, like drought, salinity, and heat, frequently coincide with biotic pressures such as pests and diseases, resulting in complex interactions that may be either synergistic or antagonistic. Understanding these relationships is essential for creating resilient crop kinds and guaranteeing food security throughout climate change. Plants have developed intricate molecular defense mechanisms to withstand concurrent abiotic and biotic stressors. These encompass transcription factors and kinases that govern integrative plant responses (Menéndez and Tundo, 2024). Drought stress, for instance, induces morphophysiological changes such as reduced leaf area and enhanced root growth, which can prime plants for future stress events. Biochemical responses include the production of secondary metabolites and volatile organic compounds, which play roles in signaling and defense against herbivores (Shafi et al., 2024). Genome editing technologies offer precise tools for enhancing plant resilience to both abiotic and biotic stresses by enabling targeted alterations in plant genomes (Mann et al., 2024). Integrating bioinformatics and AI applications can enhance the identification and analysis of stress-responsive genes and regulatory networks, providing deeper insights into stress response pathways (Zhang et al., 2024). However, multi-omics approaches, including genomics, transcriptomics, and metabolomics, are crucial for understanding the complex regulatory networks involved in stress adaptation. Combining these approaches with eco-physiological assessments can provide a comprehensive understanding of how plants adapt to changing environments (Shafi et al., 2024). Comprehending the impact of these cumulative stresses on plants at physiological, molecular, and ecological levels is crucial for cultivating crops capable of sustaining output under realistic growing conditions, where numerous stresses frequently occur.

#### 2.2.1 Interactions between salinity, drought, heat, pests, and diseases

Abiotic stresses such as salinity, drought, and heat frequently intensify the effects of biotic pressures by modifying plant physiology, hence increasing their vulnerability to pests and diseases. Salt stress can diminish plant vigor and trigger water stress, hence impairing the plant’s innate defense mechanisms against diseases such as fungi or bacteria (Shafi et al., 2024). Drought and heat can also hinder a plant’s capacity to synthesize secondary compounds, essential for protection against insect pests (Shafi et al., 2024). Elevated temperatures can compromise disease resistance, making plants more susceptible to infections from fungi and bacteria (Shelake et al., 2024).

**Figure 2:**
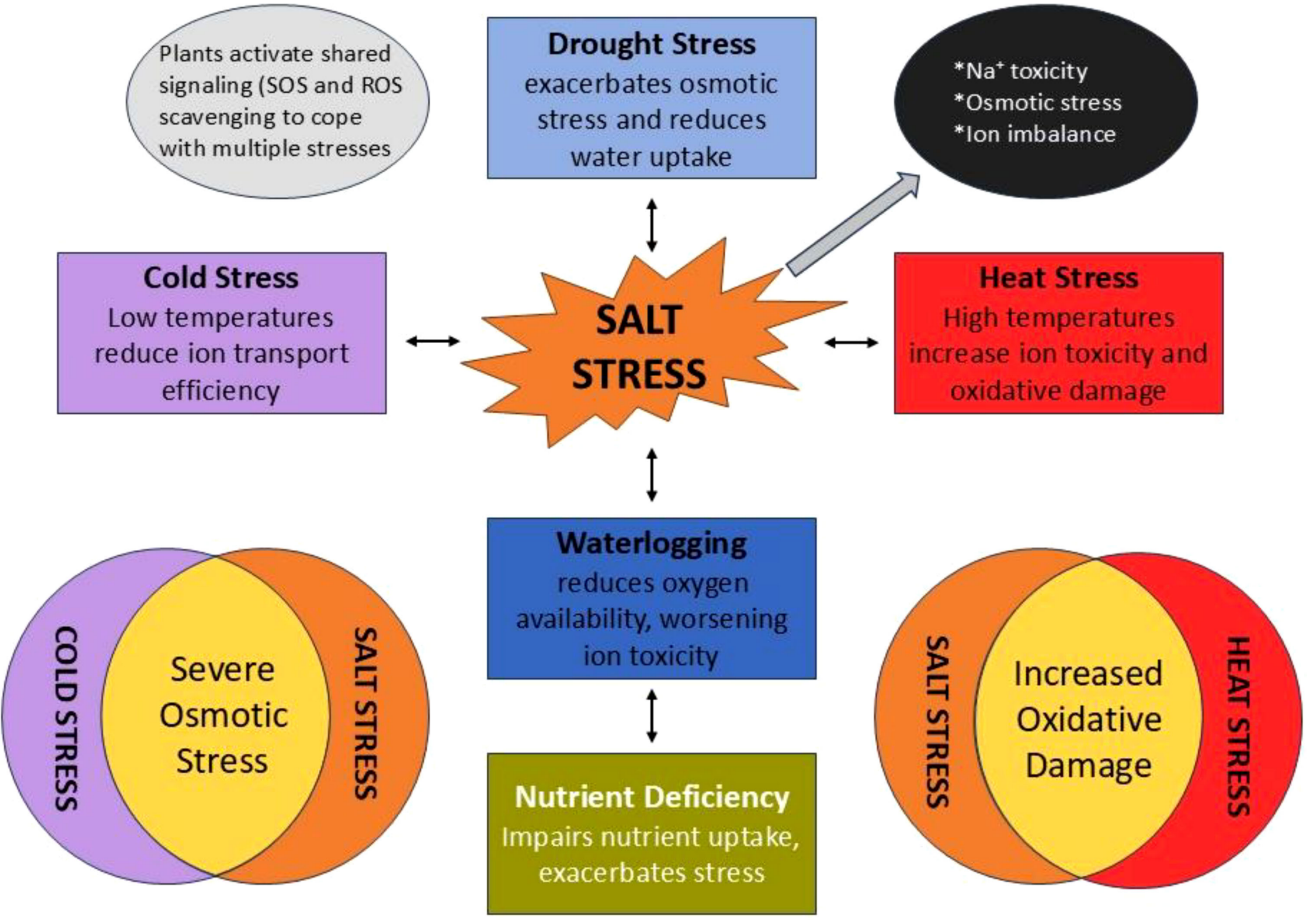
Summary of interaction between salt stress and other environmental stresses.

Conversely, the existence of pests might affect a plant’s resilience to abiotic pressures. Herbivore-induced stress can alter plant metabolism, thereby augmenting or impairing the plant’s resilience to other stressors. Research indicates that plants subjected to simultaneous drought and insect herbivory stress demonstrate modified hormonal responses, including heightened production of jasmonic acid, which is crucial to both drought resistance and pest defense (Khan et al., 2024; Margay et al., 2024). As illustrated in **Figure 2**, the combined stresses frequently have a synergistic or additive effect, whereby one stress amplifies the adverse impact of another, resulting in a more significant decline in growth, yield, and salt tolerance (Khalid et al., 2024; Li et al., 2024). In order to ensure the stability of salt tolerance characteristics under varying environmental conditions, breeding programs must integrate multi-stress tolerance. Multi-environment trials (METs) are crucial for evaluating salt-tolerant rice cultivars under diverse climatic circumstances and across several growth seasons. Such trials assist in identifying cultivars that sustain constant performance despite varying environmental conditions (Balasubramanian). Moreover, multi-stress breeding is essential to guarantee that rice varieties exhibit tolerance not only to salt but also to combined stresses such as drought and heat. This underscores the necessity for a comprehensive strategy in breeding for multi-stress tolerance, wherein the interconnections between abiotic and biotic stressors are concurrently addressed.

#### 2.2.2 Physiological and molecular responses to combined stresses

When plants suffer combined abiotic and biotic challenges, their physiological responses become more intricate than those experienced under singular stress conditions. The initial line of defense often entails a series of signaling pathways triggered by the perception of stress. In response to abiotic conditions including salt and drought, plants initiate osmotic regulation and antioxidant defense mechanisms to mitigate damage from reactive oxygen species (Liu et al., 2024; Rafique et al., 2024). Simultaneously, when plants encounter biotic stress, such as pathogen invasion, they activate defense mechanisms, including the synthesis of pathogenesis-related proteins and the initiation of systemic acquired resistance (SAR) pathways (Bandyopadhyay et al., 2024). Under conditions of combined salinity and pathogen stress, plants may prioritize their response to salinity by activating ion transporters such as NHX (Na+/H+ antiporters) and SOS (salt excessively sensitive) pathways, which facilitate the maintenance of ionic equilibrium (Maach et al., 2024). This may restrict the energy allocated for activating defense mechanisms against pathogens, leading to heightened disease vulnerability (Majidian and Ghorbani, 2024). In contrast, other plant species, such as those in the Brassica genus, exhibit cross-tolerance mechanisms that boost their response to abiotic challenges, including drought or heat, when subjected to biotic pressures like herbivore attacks (Cantila et al., 2024; Yoo et al., 2024). The interaction among various stress responses frequently entails communication between plant hormones at the molecular level. The hormone abscisic acid (ABA) is essential for drought tolerance and pathogen defense; however, the precise regulation of ABA signaling is vital in deciding whether a plant will initiate an effective defense response or prioritize survival during drought or heat stress (Rizhsky et al., 2004; Bharath et al., 2021; Lim et al., 2022). This crosstalk engenders a complex environment for breeding strategies, as selecting for tolerance to one stress may unintentionally diminish the plant’s capacity to withstand another.

#### 2.2.3 Multi-stress tolerance breeding strategies

Considering the intricate nature of combined stress reactions, breeding for multi-stress tolerance necessitates a more comprehensive strategy than conventional single-stress breeding. A variety of breeding strategies have developed to tackle the challenges posed by coupled stressors. These include Quantitative Trait Locus (QTL) Mapping and Marker-Assisted Selection (MAS), which have emerged as viable methods in recent years. For instance, QTLs linked to simultaneous drought and heat tolerance in Oryza sativa have been identified and utilized in marker-assisted selection to create varieties with improved resilience to both extremes concurrently (Hassan et al., 2023; Mohanavel et al., 2024). Transgenic and genome-editing approaches also play a significant role. Editing genes associated with ion transport, hormone control, and reactive oxygen species scavenging can result in the creation of plants that demonstrate improved tolerance to both abiotic conditions (e.g., salt, drought) and biotic challenges (e.g., diseases, pests) (Li et al., 2022; Yadav et al., 2023). The CRISPR/Cas9 technology has been employed to modify the OsERF48 gene in rice, which is involved in the regulation of drought tolerance and resistance to the rice blast fungus (Shim et al., 2018; Jung et al., 2021). Recent breakthroughs in functional genomics and systems biology have enabled researchers to examine the integrated responses of plants to various stimuli at the transcriptomic and proteomic levels. By comprehending the systemic coordination of stress responses in plants, breeders might formulate ways to augment the overall resistance of crops against various combined stressors. The utilization of transcriptome data has facilitated the identification of genes associated with heat and pest resistance in maize, potentially aiding in the breeding of maize varieties capable of enduring both stressors (Xue et al., 2024; Zhao et al., 2024).

Traditional breeding techniques remain essential in the creation of multi-stress tolerant cultivars. These programs must prioritize the assessment of stress tolerance under various environmental settings, mimicking the impacts of mixed abiotic and biotic stressors. Breeding projects for wheat have aimed to create varieties that exhibit tolerance to both drought and pest infestations, seeing some success in areas susceptible to these challenges (Tadesse, 2021; Bapela et al., 2022). Addressing combined abiotic and biotic stresses is crucial for crop resilience in the face of climate change and insect challenges. These stresses cause complicated physiological and molecular responses, typically requiring cross-talk between stress signaling pathways. Breeding efforts must use QTL mapping, transgenic methods, and functional genomics to create stress-resistant crops. Successful multi-stress tolerance breeding requires a profound understanding of plant stress physiology and a dedication to establishing breeding programs that analyze numerous stress combinations to create resilient, high-yielding crops.

## 3 Mechanisms of salinity tolerance in rice

### 3.1 Ionic homeostasis

**Figure 3:**
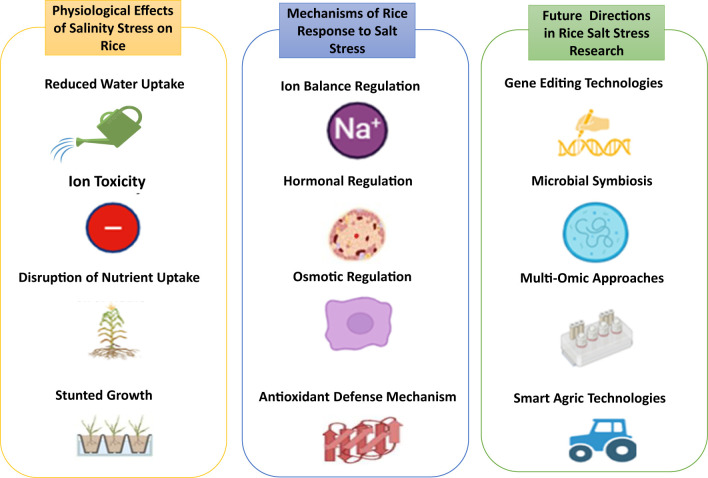
Overview of the physiological response of rice to salinity and drought stress: effects, mechanisms and future research directions.

A key mechanism enabling rice to withstand salinity is ionic homeostasis, which maintains the delicate ion balance within plant cells under saline conditions. Plant growth and productivity are significantly hampered by ionic toxicity, osmotic stress, and nutritional imbalance caused by high concentrations of sodium (Na^+^) and chloride (Cl^-^) ions in saline soils. As summarized in **Figure 3**, rice plants have developed several adaptive methods to lessen these negative effects and guarantee survival in the face of saline stress. A fundamental mechanism includes the management of sodium and potassium concentrations, in which effective ion transport networks uphold Na^+^/K^+^ balance, essential for enzyme functions and cellular integrity. The high-affinity potassium transporter (HKT) gene family, especially OsHKT1;5, is crucial for selectively extracting Na^+^ from the xylem, thus mitigating harmful buildup in the shoots while maintaining sufficient potassium levels in the cytoplasm (Shohan et al., 2019; Hussain et al., 2022). The sequestration of sodium ions into vacuoles is mediated by vacuolar Na^+^/H^+^ antiporters such as NHX1, which mitigate cytosolic toxicity and assist in maintaining osmotic equilibrium. The sequestration process is facilitated by proton pumps such as H^+^-ATPase and H^+^-PPase, which sustain the electrochemical gradient essential for Na^+^ transport (Patel and Mishra, 2021; Mansour, 2023). These physiological responses are molecular mechanisms like the salt overly sensitive (SOS) pathway, involving key components such as SOS1, SOS2, and SOS3. This pathway modulates Na^+^ efflux and facilitates K^+^ retention, hence maintaining ion homeostasis during salt stress. Transcriptomic analyses reveal the overexpression of these transporters in salt-tolerant rice cultivars, emphasizing their significance in salinity resilience (Hussain et al., 2022). These interconnected methods and mechanisms illustrate rice plants’ complex approach to sustaining ionic equilibrium and flourish in saline conditions.

Although sodium toxicity frequently generates greater focus, the management of chloride ions is of equal significance. Rice plants efficiently regulate Cl^−^ absorption and translocation to prevent excessive buildup in sensitive tissues, with stronger discrimination mechanisms facilitating improved salinity tolerance (Sarangi et al., 2024; Gupta and Shaw, 2021; Guo et al., 2024). Alongside these intrinsic tactics, the utilization of biostimulants has surfaced as a promising method. Exogenous compounds like gallic acid and humic acid improve ionic homeostasis by decreasing Na^+^ buildup and optimizing the Na^+^/K^+^ ratio. These biostimulants also stimulate antioxidant defense mechanisms, alleviating oxidative damage caused by saline environments (Rahman et al., 2022; Abu-Ria et al., 2023; Zhang et al., 2024a).

### 3.2 Osmotic adjustment

Osmotic adjustment is essential for rice plants to adapt to salinity stress, as it helps maintain cellular turgor and water uptake, both of which are crucial for survival in salt-affected environments. This adaptive mechanism relies on the active accumulation of osmolytes, including proline, glycine betaine, and trehalose, as well as inorganic ions such as potassium (K^+^). These molecules reduce cellular osmotic potential, facilitating water retention in the presence of external osmotic imbalances due to elevated salinity (Suprasanna et al., 2016; Zouari et al., 2019; Mohammadi Alagoz et al., 2023). In addition to water regulation, these osmolytes play a role in stabilizing cellular proteins and membranes, reducing ionic toxicity from Na^+^ and Cl^-^ accumulation, and enhancing antioxidant defenses. Proline serves as a molecular chaperone and a scavenger of reactive oxygen species (ROS), protecting cellular structures during stress conditions (Rehman et al., 2021; Liu et al., 2022; Ulrich, 2023). The importance of osmotic adjustment is further highlighted by research on salinity-tolerant rice lines, in which certain quantitative trait loci (QTLs) augment osmolyte synthesis and transport. These genetic modifications highlight the relevance of essential genes, especially those associated with tetrapyrrole production, which enhance osmotic balance and photosynthetic efficiency in saline environments (Nounjan et al., 2020). These findings highlight the interaction between metabolic pathways and stress resilience in rice. Advancements in biotechnology, have transformed approaches to improve osmotic adjustment. Researchers have engineered genes involved in osmolyte production to develop transgenic rice types with enhanced salt tolerance. The overexpression of glycine betaine biosynthetic genes has resulted in rice plants exhibiting enhanced growth and productivity in saline soils (Liu et al., 2022; Mandal et al., 2022; Yu et al., 2022). These insights and improvements collectively underscore the complex mechanisms of osmotic adjustment and its significant promise in developing salt-tolerant rice cultivars (Nurbekova et al., 2024).

### 3.3 Antioxidant defense

Salinity stress in rice plants induces the excessive generation of reactive oxygen species (ROS), such as superoxide anions, hydrogen peroxide (H_2_O_2_), and hydroxyl radicals, leading to oxidative damage to critical cellular components, including lipids, proteins, and DNA. Rice plants employ a comprehensive antioxidant defense system consisting of enzymatic and non-enzymatic components to preserve cellular integrity during stress. Enzymatic antioxidants, including superoxide dismutase (SOD), catalase (CAT), ascorbate peroxidase (APX), and glutathione reductase (GR), are upregulated in response to salinity stress. This increased expression establishes a defense network that transforms harmful reactive oxygen species (ROS) into less reactive molecules, such as water and oxygen. Enhanced activities of APX and CAT have been observed in salt-tolerant rice varieties, such as CSR36, leading to reduced ROS levels and improved ionic homeostasis (Li et al., 2023; Kumar et al., 2024). Non-enzymatic antioxidants such as ascorbate (AsA), glutathione (GSH), proline, and phenolic compounds serve to enhance enzymatic defenses. Proline facilitates osmotic adjustment, stabilizes proteins, and scavenges free radicals, enhancing stress tolerance (Hadid et al., 2023; Renzetti et al., 2024). SOD serves as the initial line of defense, facilitating the conversion of superoxide radicals into oxygen and hydrogen peroxide through the process of dismutation. In rice, various isoforms of superoxide dismutase (SOD), such as Cu/Zn-SOD, Mn-SOD, and Fe-SOD, are distributed across distinct cellular compartments, including chloroplasts, mitochondria, and the cytosol, to offer protection that is specific to each compartment (Sanyal et al., 2022; Zhou et al., 2022). CAT is essential for the detoxification of hydrogen peroxide, facilitating its conversion into water and oxygen. Rice cultivars demonstrating elevated CAT activity in response to salinity stress show increased tolerance, as indicated by diminished oxidative damage and enhanced growth (Jiang et al., 2023). POD and APX play crucial role in the ascorbate-glutathione cycle, facilitating the detoxification of hydrogen peroxide within the cytosol and chloroplasts. APX specifically employs ascorbate as a reducing agent to transform H_2_O_2_ into water, whereas POD plays a crucial role in lignin biosynthesis and the fortification of cell walls during stress conditions (Foyer and Kunert, 2024; Yoshimura and Ishikawa, 2024).

The application of exogenous antioxidants, including gallic acid, significantly bolsters this defense mechanism by augmenting the enzymatic activity within the AsA-GSH cycle, which in turn mitigates oxidative damage in rice seedlings subjected to salinity stress (Rahman et al., 2020). In the realm of genetics, genes like OsAPX1 and OsCATC frequently exhibit upregulation in rice varieties that demonstrate salt tolerance. Furthermore, genetic modifications, particularly the overexpression of genes responsible for scavenging reactive oxygen species, have proven effective in enhancing salinity tolerance (Chen and Qiu, 2023; Song et al., 2024; Zhao et al., 2024). Breeding programs incorporating molecular tools to enhance antioxidant pathways are essential for developing rice varieties with improved salinity tolerance. This highlights the variability of antioxidant responses among different genotypes and their significance for sustainable agriculture.

### 3.4 Morphological and anatomical adaptations

Rice plants have developed a range of morphological and anatomical adaptations that enable them to cope with the detrimental impacts of salinity stress, such as decreased water availability, ionic toxicity, and oxidative stress. The adaptations are crucial for fostering growth and ensuring productivity in saline environments. A significant approach involves altering root morphology and architecture, which improves the absorption of water and nutrients while minimizing sodium buildup. Salt-tolerant rice varieties generally exhibit deeper and more expansive root systems, allowing them to reach less saline water located at greater depths.

Alterations in the density of root hairs and heightened root exudation play a significant role in enhancing ionic balance (Oburger et al., 2014; Holz et al., 2018). In light of the diminished water availability linked to salinity, rice plants demonstrate reduced leaf areas and heightened leaf rolling, adaptations that serve to conserve water effectively. The regulation of stomata is essential in this context, as the closure of these structures reduces water loss and inhibits the absorption of harmful ions through evapotranspiration (Raeisi Vanani et al., 2024). Salt-tolerant varieties exhibit anatomical adaptations such as thicker cuticles and reinforced epidermal layers. These features minimize water loss and serve as barriers to ion entry, thereby improving the plant’s capacity to sustain internal water potential and ionic balance. Moreover, vascular adaptations, including decreased xylem vessel diameters and a heightened number of xylem vessels, play a crucial role in regulating ion transport and mitigating sodium translocation to the shoots. Improved vascular compartmentalization isolates toxic ions, thus safeguarding metabolic processes (Pouzoulet et al., 2020). Furthermore, forming aerenchyma tissue in the roots enhances oxygen transport in saline, waterlogged soils, offering significant advantages for lowland rice varieties. Ultimately, the buildup of osmolytes such as proline and glycine betaine within the leaves and stems of rice plants plays a crucial role in sustaining cell turgor and osmotic potential. This adaptation allows the plants to persist in growth and photosynthesis, even when faced with salinity stress. The various adaptations contribute significantly to the resilience of salt-tolerant rice varieties, offering important insights for breeding programs and agricultural practices focused on enhancing rice production in areas susceptible to salinity.

## 4 Genetics of salinity tolerance in rice

### 4.1 Key quantitative trait Loci

Salinity tolerance in rice is modulated by complex genetic systems, with many Quantitative Trait Loci (QTLs) essential for sustaining ionic homeostasis, osmotic equilibrium, and stress signaling pathways. Recent advances in genomic technologies have facilitated the precise identification of key QTLs associated with salinity tolerance, especially during crucial growth phases. The genetic complexity of these features renders them difficult to identify and enhance via conventional breeding techniques. Modern methodologies such as Quantitative Trait Locus (QTL) mapping and Genome-Wide Association Studies (GWAS) have facilitated the identification of loci linked to salinity tolerance. Mapping these loci enhances the understanding of the genetic variation that contributes to salt tolerance in rice, hence informing breeding initiatives (Yin et al., 2023; Sugasi et al., 2024). One of the most thoroughly investigated QTLs is Saltol, situated on chromosome 1, which is significantly linked to the regulation of sodium-potassium homeostasis during salt stress (Marè et al., 2023). Saltol accounts for 62–80% of phenotypic variation under salinity stress (Nutan et al., 2019; Marè et al., 2023). The Saltol region, especially the SKC1 gene responsible for encoding a sodium-potassium transporter, has played a crucial role in marker-assisted selection (MAS) initiatives aimed at creating salt-tolerant rice varieties, exemplified by FL478. Research on haplotypes has demonstrated that various alleles in this region play a significant role in salinity tolerance, observed in elite lines and various landraces (Marè et al., 2023; Thippani et al., 2023). A comprehensive meta-analysis focusing on salinity tolerance QTLs has successfully identified 65 meta-QTLs (mQTLs) derived from an initial pool of 768 QTLs across 35 studies. This extensive research has effectively refined the confidence intervals for various traits, encompassing root architecture, osmotic adjustment, and ionic transport. Identifying these mQTLs has emerged as a critical focus for breeding initiatives designed to enhance salt tolerance (Satasiya et al., 2024). Recent sequencing studies have brought to light the significance of qCMS1 and qTN1 located on chromosome 1, which are linked to important traits such as cell membrane stability and tiller number in the context of salt stress. The identified loci exhibit notable phenotypic variance ranging from 16% to 20%, positioning them as strong candidates for the pyramiding of multiple QTLs aimed at improving salt tolerance (Songtoasesakul et al., 2023; Khunsanit et al., 2024).

Moreover, genome-wide association studies (GWAS) have identified new quantitative trait loci (QTLs), such as qGPR2 and qSLR9, which regulate seedling development and germination capacity in saline environments, providing new avenues for genetic enhancement in breeding programs employing SNP markers and marker-assisted selection (MAS) (Sayed et al., 2022; Baştaş, 2023; Maniruzzaman et al., 2023). The genes associated with these QTLs encompass transcription factors (e.g., MADS-box, calmodulin-binding proteins) and ion transporters (e.g., OsHKT1;5, OsSOS1), which are pivotal in ion transport, signaling, and stress responses, elucidating the molecular mechanisms of salinity tolerance (Leawtrakun et al., 2024a). Incorporating these quantitative trait loci into superior rice varieties, along with marker-assisted selection and advanced gene-editing technologies, are facilitating the creation of rice cultivars that exhibit improved tolerance to salinity. The recent advancements hold considerable importance for global food security, facilitating more sustainable rice production in areas susceptible to salinity, thus contributing to the stabilization of yields in the face of escalating environmental challenges.

### 4.2 Molecular markers, marker-assisted selection, and modern breeding approaches for salt tolerance

In recent years, molecular markers and marker-assisted breeding (MAB) have become essential instruments for improving the salinity stress tolerance of rice varieties. Aside the Saltol QTL on chromosome 1, recent investigations have revealed additional QTLs, including qSES1, qK8, and qRL1, which exhibit distinct placements and notable stability across various genetic backgrounds, offering new opportunities for developing salt-tolerant rice varieties (Maniruzzaman et al., 2023). Molecular markers, such as single nucleotide polymorphisms (SNPs) and random amplified polymorphic DNA (RAPD) markers, have greatly enhanced the high-resolution mapping of salt-tolerant traits. SNP markers are crucial for accurately identifying traits in salt-tolerant rice, whereas RAPD markers are effective for screening aromatic rice varieties (Cuthbertson, 2022; Geng et al., 2023; Maniruzzaman et al., 2023). Marker-assisted backcrossing (MABC) effectively transfers salinity tolerance traits into elite varieties. Examples of success include the introgression of the Saltol locus into temperate japonica rice varieties such as *Vialone Nano* and *Onice*, which improves salinity tolerance while maintaining yield potential (Marè et al., 2023). In addition to Saltol, loci including SKC1 and new QTLs derived from landraces, such as *Akundi*, provide supplementary mechanisms for salinity tolerance, thereby expanding the genetic resources accessible for breeding purposes (Soda et al., 2013; Le et al., 2021). The variety CSR43, produced using MABC, has exhibited high yields under saline environments in India (Singh et al., 2016). Likewise, BRRI dhan67, introduced in Bangladesh, has demonstrated exceptional adaptation to coastal areas characterized by elevated soil salinity (Shamsuddin et al., 2022). These achievements underscore the promise of molecular markers in the creation of climate-resilient rice cultivars.

Omics technologies, including as transcriptomics and proteomics, have detailed the molecular pathways that govern salinity tolerance. Transcriptomic analyses have demonstrated the overexpression of genes like OsDREB2A and WRKY53, which are pivotal in stress response pathways. Proteomic investigations have discovered stress-protective proteins that facilitate osmotic adjustment and mitigate oxidative stress, thereby deepening the understanding of salt tolerance processes (Reddy et al., 2023). These developments have facilitated the precise modification of genes such as OsHKT1;5 and OsNHX1, which govern Na^+^ transport and vacuolar sequestration, respectively, enhancing salinity tolerance without diminishing yield (Rasheed et al., 2022). Modern breeding approaches, notably CRISPR/Cas9-mediated genome editing, genomic selection, and high-throughput phenotyping, have enhanced traditional and marker-assisted breeding procedures. For instance, CRISPR/Cas9 has been employed to delete the OsERF922 gene, thereby augmenting blast resistance in rice while preserving other agronomic characteristics (Liang et al., 2021). Similarly, genomic selection (GS) has been utilized to enhance complex traits, including grain quality and nitrogen use efficiency (NUE), in rice (Yu et al., 2022). High-throughput phenotyping platforms, coupled with advanced imaging technologies, have enabled the rapid screening of large breeding populations for traits such as canopy architecture, root morphology, and stress responses (Yang et al., 2014; Tanger et al., 2017). The combined use of genetic approaches (e.g., GWAS, GS) with high-throughput phenotyping has enabled the creation of salinity-tolerant rice cultivars with unparalleled precision and efficiency (Akhlasur Rahman et al., 2024).

### 4.3 Genomic insights

The utilization of genomic technologies has significantly enhanced the study of salt tolerance in rice, uncovering complicated biochemical and genetic pathways that regulate this feature. Candidate genes such as MIKC-type MADS domain proteins and calmodulin-binding transcription factors have been emphasized for their involvement in salt stress signaling (Leawtrakun et al., 2024a). Further insight from transcriptomics and eQTL analyses reveals that salinity induces significant changes in gene expression in rice. The studies indicate that trans-eQTLs have a greater impact compared to cis-eQTLs, implying the importance of master regulatory genes in the adaptation to salinity stress (Gupta et al., 2024). Moreover, transcriptomic analysis highlights the potential of spermidine treatment in mitigating salinity-induced transcriptional disruptions, upregulating genes related to stress-alleviation pathways such as MAPK signaling and phenylalanine metabolism, thus complementing genomic strategies for enhanced salinity tolerance (Shen et al., 2024). Simultaneously, advanced genotyping technologies such as SNP chips and next-generation sequencing (NGS) have significantly propelled genome-wide association studies (GWAS) and marker-assisted selection (MAS) aimed at enhancing salinity tolerance. These instruments facilitate the recognition of markers associated with salinity, thus empowering more accurate breeding initiatives aimed at multi-trait tolerance (Singh and Devi, 2023). The exploration of functional genomics, especially regarding genes like OsHKT1;5 and OsNHX1, has deepened our understanding of the intricate mechanisms involved in ion transport and compartmentalization when faced with salinity stress.

### 4.4 Role of wild rice germplasm

Wild rice germplasm has become crucial for improving salinity tolerance in cultivated rice cultivars, tackling a significant agricultural concern. The genetic variety in wild rice species offers unique alleles and features that markedly enhance tolerance to salt stress, exceeding the constraints observed in domesticated varieties such as *Oryza sativa* (Shahzad et al., 2022). Species such as *Oryza rufipogon*, *Oryza coarctata*, *Oryza latifolia*, and *Oryza alta* are naturally adapted to saline environments, equipped with mechanisms including effective Na^+^ exclusion, K^+^ retention, and osmotic adjustment. These wild cousins offer distinct genes responsible for activities such as vacuolar sequestration of sodium, regulated xylem loading, and activation of stress-responsive antioxidant pathways, all contributing to salt tolerance (Solis et al., 2020). Furthermore, metabolomic analysis of Dongxiang wild rice has shown the important function of amino acids such as L-Asparagine in reducing salt stress and offering biochemical markers for tolerance building. Crucially for preserving cellular homeostasis under salt stress conditions, these metabolic changes include the activation of antioxidant pathways and the buildup of osmolytes (Chen et al., 2021a). Moreover, the identification of new Quantitative Trait Loci (QTLs) and candidate genes such as AGO2 and WRKY53, which improve salt tolerance at crucial phases like germination and early development, has been much aided by the establishment of Chromosome Segment Substitution Lines (CSSLs). The key to stress adaption is that these genes control fundamental processes, including reactive oxygen species (ROS) scavenging and ion transport (Xing et al., 2024). A number of studies have effectively pinpointed and described salt-tolerant genes in wild rice species, while current endeavors focus on incorporating these traits into superior rice cultivars, thereby enhancing the genetic diversity accessible for salinity tolerance (Samy et al., 2024).

## 5 Physiological and biochemical responses to salinity

### 5.1 Impacts on photosynthesis and chlorophyll content

Salinity stress significantly impairs essential processes of photosynthesis and chlorophyll concentration in *O*. *sativa*, which are vital for plant growth and yield. Sodium ions (Na^+^) interfere with cellular ionic equilibrium and osmotic potential, causing structural and functional damage to the photosynthetic system. This disturbance results in diminished photosynthetic efficiency, decreased chlorophyll levels, and impaired light absorption. Salinity inhibits chlorophyll biosynthesis and hastens its breakdown, reducing light absorption and electron transport. A study on rice cultivars subjected to 80 mM NaCl demonstrated considerable chlorophyll decline, chiefly attributable to oxidative damage and the suppression of enzyme pathways essential for chlorophyll biosynthesis (da Silva et al., 2023). Rice genotypes possessing salt-tolerant QTLs, exemplified by the Pokkali variety, exhibited improved chlorophyll retention and superior photosynthetic efficiency in saline conditions, highlighting the significance of genetic diversity in stress adaptation (Nounjan et al., 2020). Furthermore, salt stress affects chloroplast thylakoid membranes, limiting Photosystem II (PSII) activity. This leads to reduced quantum efficiency (Fv/Fm) and poorer photochemical quenching, both of which are important for effective energy conversion during photosynthesis (Tabassam et al., 2016). Salt-tolerant genotypes, however, preserve higher PSII efficiency, ensuring better utilization of light energy. As a result, the overall photosynthetic rate (Pn) drops during salinity stress, mostly due to stomatal closure and lower CO_2_ absorption. Improvements in water use efficiency (WUE) and osmotic adaptations in salt-tolerant cultivars alleviate these impacts, as demonstrated by Pokkali rice, which showed a 19% rise in Pn under high salinity, indicating its superior physiological resilience (Santanoo et al., 2023).

**Figure 4:**
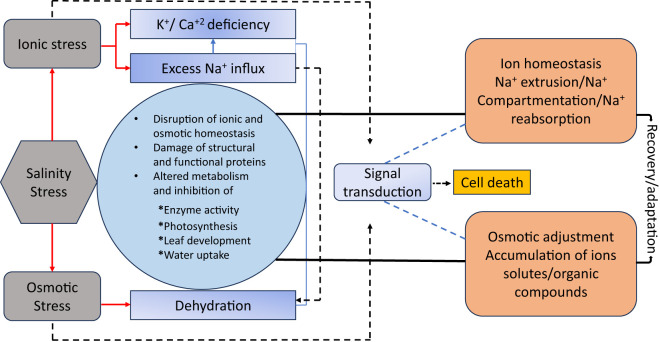
Illustrative summary of the physiological response of rice to salinity stress.

**Figure 4** illustrates that physiological alterations under salinity stress encompass various plant responses that result in diminished growth (Riaz et al., 2019). These changes include alterations in the Na+/K+ ratio, decreased stomatal conductance (gs), reduced photosynthetic rates, and heightened production of reactive oxygen species (ROS) to mitigate the detrimental effects of salinity on photosynthesis and to stimulate the synthesis of antioxidant enzymes. Moreover, antioxidant enzymes and osmoprotectants, like glutathione and proline, are crucial in safeguarding the photosynthetic apparatus from oxidative damage caused by salinity. These chemicals neutralize reactive oxygen species (ROS), mitigating cellular damage and preserving chloroplast integrity. Genetic findings regarding salt adaptation emphasize the significance of chlorophyll biosynthesis genes, particularly Os08g41990, which have demonstrated the ability to sustain chlorophyll levels under stress conditions. Studies employing chromosomal segment substitution lines (CSSLs) have demonstrated the significance of these genes in improving the rate of photosynthesis and strengthening salt tolerance (Nounjan et al., 2020). Thus, physiological and genetic techniques are required to minimize salinity’s deleterious effects on rice’s photosynthesis.

### 5.2 Changes in nutrient uptake and transport

By mainly disrupting ionic equilibrium and nutritional shortages, salinity stress greatly hinders nutrient uptake and transport in rice. Key macronutrients like potassium (K^+^), calcium (Ca^+^), and magnesium (Mg^2+^), and micronutrients like manganese (Mn) and zinc (Zn) are hindered in their uptake by salty environments’ high sodium (Na^+^) and chloride (Cl^−^) concentrations. Ionic imbalances hinder plant development and productivity by interfering with essential physiological processes, including photosynthesis and enzymatic activities. Salinity significantly disturbs sodium-potassium homeostasis by elevating the Na^+^/K^+^ ratio, hence impairing metabolic processes and enzymatic functioning. Nevertheless, several salt-tolerant rice cultivars, including Pokkali, alleviate these impacts by effectively regulating Na^+^ and K^+^ transporters, such as OsHKT1;5 and OsNHX1, which decrease Na^+^ buildup in the shoots while preserving sufficient K^+^ levels for cellular activities (Nampei et al., 2021). Wild rice species, including *O*. *coarctata*, demonstrate mechanisms that improve K^+^ retention and mitigate Na^+^ toxicity through effective xylem unloading and vacuolar sequestration (Sarkar and Roy, 2020). Moreover, salt stress impedes the absorption of micronutrients, especially manganese and zinc, which are crucial for photosynthesis and enzymatic activities. Supplementing manganese under salty conditions aids in restoring nutritional equilibrium and promotes stress tolerance, presenting a viable technique for increasing rice development under salinity (Tabassam et al., 2022).

Calcium (Ca^2+^) is essential in decreasing Na^+^ toxicity by restricting Na^+^ transport to aerial parts and enhancing Cl^−^ exclusion. In salt-tolerant cultivars like Nona Bokra, appropriate calcium levels considerably boost survival rates under salinity by reducing Na^+^ and Cl^−^ translocation (Abouzied and Abd El-latif, 2017). Furthermore, elevated rhizospheric pH in saline environments restricts iron accessibility (Fe), an essential nutrient for plant development. Salinity-tolerant genotypes mitigate this issue by enhancing the expression of Fe transporters, including OsIRT1 and OsYSL15, so securing sufficient Fe availability in saline-alkaline environments (Nampei et al., 2021). The symbiosis between arbuscular mycorrhizal (AM) fungus and rice is another molecular process that contributes to salt tolerance. By controlling the genes for cation transporters, AM fungi improve root potassium absorption and root sodium compartmentalization, which in turn reduces root-to-shoot Na+ transit. Another way to increase rice’s tolerance to salt is through this interaction, which upregulates genes such as OsSOS1 and OsNHX3 that help maintain ionic balance when the plant is exposed to salt (Porcel et al., 2016).

### 5.3 Hormonal regulation

Salinity stress in *O*. *sativa* produces complicated hormonal changes that play a critical role in minimizing the detrimental consequences of excessive salt concentrations. Hormonal signaling serves as a conduit, merging environmental stimuli with growth and stress response pathways, allowing rice plants to acclimate to salinity stress via processes such as stomatal control, ionic homeostasis, and mitigation of oxidative damage (Xiao and Zhou, 2023). Elevated salt concentrations stimulate the excessive generation of reactive oxygen species (ROS), resulting in oxidative damage to plant tissues, including lipid peroxidation, protein breakdown, and DNA damage. Malondialdehyde (MDA), a byproduct of lipid peroxidation, functions as a measure for oxidative stress levels in plants (Zhao et al., 2021; Zhao et al., 2024). Spermidine (Spd) treatment enhances the activity of antioxidant enzymes such as superoxide dismutase (SOD) and catalase (CAT), significantly reducing reactive oxygen species (ROS) levels and boosting oxidative stress tolerance, especially in rice roots (Shen et al., 2024).

Central to the salinity stress response is abscisic acid (ABA), frequently referred to as the “stress hormone.” ABA concentrations increase significantly during salt stress, inducing stomatal closure to diminish transpiration and avert excessive water loss (Xiao and Zhou, 2023). Additionally, ABA stimulates downstream signaling pathways that engage ABA-responsive element-binding factors (ABFs), which modulate stress-protective genes and enhance water uptake efficiency by improving root hydraulic conductivity (Formentin et al., 2018). Salinity stress inhibits the manufacture of auxins (IAA) and gibberellins (GA), hormones essential for cell elongation and shoot development. In salt-tolerant rice varieties, an accelerated recovery of IAA and GA levels facilitates root and shoot regrowth following initial stress (Riaz et al., 2019; Liu et al., 2022). Cytokinin levels, which decline under salinity stress, also limit cell division and shoot growth. However, higher cytokinin levels in salt-tolerant varieties, particularly in the roots, enhance nutrient translocation and stimulate shoot development (Mandal et al., 2022; Li et al., 2023). They also interact with other hormones, such as abscisic acid (ABA), to modulate stress responses and delay senescence, thereby enhancing crop yields (Yu et al., 2022). Ethylene, a hormone increased by salinity, has a dual role. It induces stress-adaptive responses like leaf senescence and aerenchyma formation, but excessive ethylene can hinder growth. Rice varieties that regulate ethylene biosynthesis and signaling enhance growth in saline conditions (Ji et al., 2020). Salicylic acid (SA) and jasmonic acid (JA) also regulate defense responses to oxidative stress and ionic imbalance. SA boosts antioxidant enzyme activity to reduce ROS damage and promotes gene expression related to programmed cell death and autophagy, facilitating stress recovery (Khan et al., 2019). These hormones interact intricately, with ABA synergistically engaging with ethylene, SA, and cytokinins to coordinate adaptive responses. For example, the ABA-ethylene synergy helps regulate stomatal dynamics, while the interplay between ABA and SA enhances ROS scavenging mechanisms (Formentin et al., 2018). These hormonal responses create a network allowing rice plants to effectively manage salinity stress.

## 6 Breeding strategies for salinity tolerance

### 6.1 Conventional breeding

**Figure 5:**
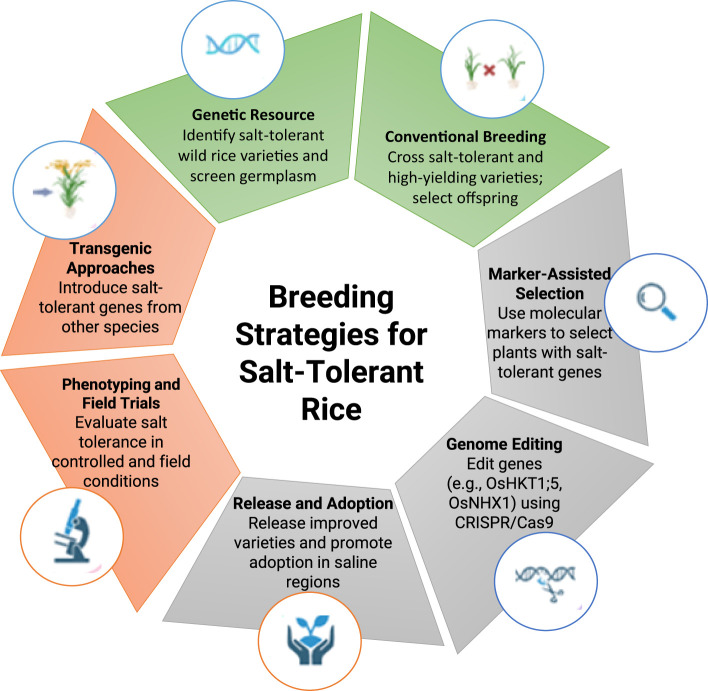
Illustrative summary of breeding strategies for salt-tolerant rice.

Traditional breeding has been essential in creating salinity-resistant rice varieties, especially in areas with restricted access to sophisticated molecular methods. Traditional breeding techniques have effectively utilized the inherent genetic variety in rice germplasm to identify and integrate features that improve salinity tolerance, providing sustainable methods to address soil salinization in prominent rice cultivation regions (**Figure 5**) (Mheni et al., 2024). Traditional salt-tolerant landraces, such as Pokkali and Nona Bokra, are among the principal contributions to these breeding initiatives and have demonstrated significant value. These kinds exhibit sodium exclusion, potassium retention, and improved osmotic adjustment, rendering them suitable donors for salinity tolerance to elite, high-yield rice cultivars (Chen et al., 2021b).

Recurrent selection, a cross-breeding approach, has been extensively utilized to impart saline tolerance to vulnerable types. By consistently selecting progeny exhibiting enhanced tolerance qualities, breeders have achieved gradual genetic improvements across generations. Crosses between Pokkali and the sensitive variety IR29 have created recombinant inbred lines (RILs) like FL478, which demonstrate enhanced salinity tolerance (Reddy et al., 2023). Phenotypic screening under controlled saline conditions has been essential for discovering salt-tolerant lineages. Parameters like seedling vigor, shoot biomass, and Na^+^/K^+^ ratios are employed to evaluate the lines’ resistance, while field testing in saline regions further corroborates the performance of these lines under natural settings (Aggarwal et al., 2024; Kumawat et al., 2024). Concerning notable accomplishments, including the creation and extensive utilization of salt-tolerant varieties such as BRRI dhan47 in Bangladesh and CSR10 in India, conventional breeding continues to encounter obstacles (Shamsuddin et al., 2022; Saminadane et al., 2024). The polygenic nature of salinity tolerance indicates that the integration of various traits into a single variety necessitates careful selection and assessment across multiple generations (Qin et al., 2020). The detailed nature of this issue restricts the pace at which salinity-tolerant varieties can be cultivated and introduced, posing a significant challenge for breeding initiatives (Akhlasur Rahman et al., 2024). Nonetheless, traditional breeding continues to serve as a fundamental strategy in tackling salinity stress in rice, providing practical and effective solutions, particularly in resource-constrained areas. Efforts to incorporate marker-assisted selection (MAS) with traditional breeding methods can speed up the process of developing better cultivars. To tackle salinity issues, it is possible to use a wider genetic base by incorporating novel donors, like wild rice species (Singh and Devi, 2023).

### 6.2 Transgenic approaches

Transgenic methods have greatly progressed the creation of salinity-resistant rice cultivars by facilitating exact genetic alterations that improve stress tolerance. These strategies aim to introduce and express particular genes from various sources to enhance rice’s ionic control, osmotic equilibrium, and antioxidative responses. For instance, the overexpression of stress-responsive genes such as OsHKT1;5 and OsNHX1 improves sodium exclusion and compartmentalization, which are essential for sustaining Na^+^/K^+^ equilibrium in saline environments (Kobayashi et al., 2017; Prusty et al., 2018). Moreover, transgenic rice, including SaPMP3 from *Spartina alterniflora* has demonstrated enhanced ion homeostasis and plant vitality under saline stress (Biradar, 2012). The functional integration of halophyte-derived genes, such as SaVHAc1 (vacuolar H^+^-ATPase subunit) from *S*. *alterniflora*, enhances this methodology. Transgenic rice, including SaVHAc1 and SaPMP3 exhibits enhanced chlorophyll retention, decreased ion toxicity, and improved grain production under high-salinity conditions (Baisakh et al., 2012; Biradar et al., 2018). Moreover, the inhibition of stress-sensitive genes via RNA interference, specifically the silencing of OsDSR2, augments antioxidant activity, diminishes oxidative damage, and elevates proline accumulation, resulting in enhanced survival rates (Mamta and Rajam, 2018; Gahlaut et al., 2021). The regulation of antioxidant enzymes, including superoxide dismutase (SOD) and catalase, enhances resilience by reducing reactive oxygen species (ROS) damage, hence maintaining cellular integrity during oxidative stress (Reddy et al., 2023).

### 6.3 CRISPR-Cas9 applications

The CRISPR-Cas9 technique has transformed the emergence of salinity-tolerant rice by facilitating accurate genome editing to target genes essential for stress response pathways, improving ionic homeostasis, antioxidant capacity, and osmotic regulation. The downregulation of OsAKT1, a gene involved in potassium and sodium transport, in the salt-sensitive IR29 rice cultivar has been accomplished via CRISPR-Cas9, resulting in diminished sodium leakage and enhanced potassium retention, thus markedly improving survival in saline environments (Khan et al., 2023). In a similar vein, the elimination of negative regulators such as OsPUB7, which inhibits salinity tolerance, has demonstrated significant promise; rice lines with null mutations in OsPUB7 displayed elevated proline levels, less ion leakage, and improved survival rates under salt stress (Kim et al., 2023). This method has been successfully utilized in hybrid rice systems, where CRISPR-Cas9 editing of OsRR22, a crucial regulator of salinity responses, produced transgene-free third-generation hybrid rice exhibiting improved salt tolerance and consistent agronomic performance (Sheng et al., 2023). Another innovative application focuses on targeting the promoter regions of salinity-associated genes, such as OsSRFP1, inside the ubiquitination pathway; altering its promoter area has refined stress control mechanisms, enhancing the rice plant’s resilience to salt stress (Linh Khanh et al., 2024). Although CRISPR-Cas9 has significant potential, its integration with other genomic technologies, such as transcriptomics and phenomics, could further improve salt-tolerance characteristics. However, problems like legislative limitations and the establishment of effective delivery methods must be confronted, with the generation of transgene-free edited lines being crucial for broad acceptability and implementation.

#### 6.3.1 Regulatory and ethical considerations for CRISPR/Cas9

The emergence of CRISPR/Cas9 technology has transformed plant breeding by facilitating precise genetic modifications with remarkable accuracy. Nonetheless, its use in agriculture presents significant regulatory, ethical, and biosafety issues that require meticulous consideration to guarantee its safe and equitable deployment. As CRISPR-edited crops approach commercialization, it is imperative to navigate the regulatory framework and address ethical considerations for their effective incorporation into global agriculture. The regulatory framework for genetically modified crops differs greatly among countries, illustrating diverse strategies for reconciling innovation with safety. In contrast to conventional genetically modified organisms (GMOs), which often entail the introduction of exogenous genes, CRISPR/Cas9 facilitates the precise alteration of an organism’s existing DNA. This differentiation has prompted continuous discussions on whether CRISPR-edited crops have to adhere to the same rigorous rules as GMOs or be classified as a unique category entirely (Prusty et al., 2018). In some countries, such as the United States, regulatory authorities like the U.S. Department of Agriculture (USDA) and the Food and Drug Administration (FDA) have embraced a more adaptable approach, proposing that CRISPR-edited crops be regulated according to their characteristics rather than the technology employed in their creation, or whether they could have been produced through conventional breeding techniques (Ahmad et al., 2023). This method facilitates expedited approval procedures for crops that do not incorporate foreign DNA. In contrast, the European Union (EU) has adopted a more prudent stance, imposing identical stringent rules on CRISPR-edited crops as those applied to conventional GMOs. This regulatory disparity presents obstacles for global markets and trade. Crops sanctioned in one jurisdiction may encounter regulatory obstacles or complete prohibitions in others. Moreover, conflicting rules may induce confusion and impede the advancement of CRISPR-edited crops, thereby postponing their advantages for farmers and consumers.

#### 6.3.2 Ethical and biosafety concerns

In addition to regulatory difficulties, CRISPR/Cas9 presents other ethical and biosafety concerns. A significant ethical problem pertains to the possibility of unexpected outcomes, such as off-target mutations or the introduction of characteristics that may yield unknown ecological effects. For instance, research shows that off-target changes may occur, where the CRISPR-Cas9 system unintentionally alters non-target genomic regions, resulting in minor insertions, deletions, or nucleotide swaps (Sturme et al., 2022). The enduring impacts of CRISPR-modified crops on biodiversity, soil integrity, and non-target species remain inadequately comprehended, necessitating comprehensive biosafety evaluations to guarantee that these crops do not inflict ecological damage. Also, there are concerns regarding the equity and accessibility of CRISPR technology persist. The capacity to patent gene-edited crops may result in monopolies over essential agricultural features, thereby centralizing the advantages of CRISPR technology within a select number of multinational firms and restricting access for smallholder farmers in underdeveloped nations (Dace and Tony Blair Institute for Global Change, 2021). This may intensify pre-existing disparities in global food systems, especially if the technology is predominantly motivated by profit rather than the public interest. Guaranteeing fair access to CRISPR technology, particularly in resource-limited environments, necessitates international collaboration and open policies.

Another ethical problem is the possibility of “designer crops” customized to particular consumer tastes. Although CRISPR has the potential to create crops with superior nutritional profiles or enhanced disease resistance, there are concerns regarding the commercialization of traits aimed at affluent consumers, potentially overlooking the requirements of deprived communities who may derive greater benefit from traits like drought resistance or disease tolerance (Adane and Alamnie, 2024; Chen et al., 2024). The public’s view and acceptability of CRISPR-edited crops significantly influence their uptake, underscoring the necessity for clear communication and public engagement in the regulatory process (Food Standards Agency, 2021). This underscores the necessity for ethical considerations in prioritizing CRISPR research and development, ensuring that the technology serves all societal sectors, especially those most susceptible to climate change and food shortages.

#### 6.3.3 The need for international collaboration

Considering the global scope of food production and trade, international cooperation is essential for creating a unified regulatory framework for CRISPR-edited crops. Harmonized international standards would ease trade obstacles, eliminate regulatory fragmentation, and promote the global use of CRISPR technology (Gan and Ling, 2022). Entities like the World Health Organization (WHO), the Food and Agriculture Organization (FAO), and the Organization for Economic Co-operation and Development (OECD) are crucial in facilitating discourse and establishing guidelines for the secure application of CRISPR in agriculture. Moreover, international collaboration might mitigate ethical problems by fostering common values and principles in the production and dissemination of CRISPR-edited crops. This entails guaranteeing an even distribution of CRISPR technology benefits and the management of any dangers through comprehensive safety evaluations and oversight. A worldwide approach would enhance the dissemination of knowledge and resources, guaranteeing that developing nations are not overlooked in the biotechnology revolution. Although CRISPR/Cas9 presents significant potential for revolutionizing agriculture, its application requires careful regulation, ethical deliberation, and global collaboration. Addressing these difficulties is essential for unlocking the complete potential of CRISPR technology while ensuring human health, environmental sustainability, and social equality.

## 7 Management practices for salinity-affected areas

### 7.1 Agronomic practices

Agronomic approaches are essential for alleviating salt stress in rice agriculture by optimizing water, soil, and nutrient management, therefore improving productivity and maintaining soil health in saline-prone areas. Efficient irrigation management is essential for regulating salt levels in the root zone. Methods like alternating wetting and drying (AWD) and integrated irrigation-drainage systems have effectively diminished salt accumulation while preserving water use efficiency. Replacing saline water every three days in fields with a ponding depth of 2-5 cm markedly enhanced the production of salt-tolerant rice varieties such as Binadhan-10 and BRRI dhan-47 (Rahman et al., 2020). Conservation agriculture approaches, such as zero tillage, crop residue recycling, and crop rotations, mitigate soil salinity, enhance organic carbon levels, and augment soil water retention capacity. Research in the Ganges Delta has shown that these methods reduce water footprints and increase profitability relative to traditional tillage systems (Sarangi et al). Effective nutrient management is essential for minimizing salinity stress. Utilizing essential nutrients like zinc and potassium via foliar sprays mitigates ionic toxicity and enhances plant vitality. Moreover, the integration of continuous saturation irrigation with organic amendments like farmyard manure and vermicompost has demonstrated a 19% increase in rice yields compared to traditional methods in the saline soils of Bangladesh (Ali et al., 2024). Soil reclamation methods improve the management of salinity. Integrating organic amendments such as green manure and gypsum diminishes soil salinity, boosting microbial activity and improving soil structure. Utilizing raised-bed cultivation with these amendments effectively reduces salt intrusion in coastal agro-ecosystems (Mitran et al., 2021). Farmer-led approaches integrating salt-tolerant varieties (STVs) with modified agronomic practices, including nutrient management specific to sodic soils, have demonstrated significant outcomes. Participatory techniques enhanced rice yields by 35% in reclaimed saline areas, highlighting the significance of farmer involvement in adopting sustainable and localized practices (Singh and Singh, 2022).

### 7.2 Crop rotation and intercropping

Sustainable soil improvement, reduced salinity accumulation, and optimal resource use efficiency can be achieved by crop rotation and intercropping agronomic procedures, which are essential for salinity management in rice agriculture. These approaches promote long-term agricultural resilience in saline-prone regions by utilizing the complimentary features of multiple crops, which mitigates salt stress. Crop rotation is essential for salinity control, as it involves using salt-tolerant species like barley or mustard, which improve water infiltration and reduce salt buildup in the root zone. This method also promotes the drainage of surplus salts, establishing a conducive soil condition for future crops. Legume rotations, including pigeon pea, augment soil organic matter and nitrogen levels, so mitigating saline effects on rice. Research on saline soils in India indicates that rice-mustard and rice-sunflower rotations enhance soil permeability, decrease salinity levels, and sustain elevated rice-equivalent yields (Mitran et al., 2021).

Intercropping enhances crop rotation by improving soil health and production. The integration of rice with salt-tolerant legumes such as mung bean or cowpea enhances soil fertility and microbial activity, alleviating the detrimental impacts of salinity. Strategic intercropping methods, such as ridge-planted pigeon peas alongside furrow-planted rice, optimize water and nutrient utilization, promote soil aeration, and enhance yield stability under saline stress (Kumar et al., 2012). Furthermore, biodiversity-based intercropping of traditional and hybrid rice varieties, as evidenced in Yunnan Province, China, enhances system resilience and stabilizes yields under saline conditions. This method reintroduces historic rice varieties and enhances genetic variety, hence establishing resilient cropping systems (http://bioscience.oxfordjournals.org/). The incorporation of these approaches with organic amendments, such as farmyard manure and green leaf manure, enhances their synergistic advantages. Improving soil organic carbon, microbial biomass, and overall productivity highlights the efficacy of integrating crop rotation, intercropping, and organic inputs for successful salt management in rice cultivation (Raeisi Vanani et al., 2024).

### 7.3 Soil reclamation

Soil reclamation in minimizing salinity stress in rice farming aims to restore the productivity of salt-affected soils by enhancing soil structure, decreasing salinity, and improving crop performance. This is accomplished by a comprehensive approach integrating physical, chemical, and biological approaches. Gypsum and phosphogypsum are efficient chemical additions for displacing sodium ions from soil exchange sites, thus mitigating sodicity and enhancing soil permeability. Studies in coastal Bangladesh indicated that the synergistic application of phospho-gypsum and cyanobacteria markedly diminished soil electrical conductivity (EC) and enhanced rice yields by 15.3% during wet seasons (Ali et al., 2023). Likewise, incorporating organic materials, including farmyard manure, compost, and biochar, enhances soil carbon levels and microbial activity. In Nigeria’s saline-sodic soils, biochar derived from rice straw and Typha grass significantly diminished salinity, enhanced water retention, and augmented rice biomass (Adam et al., 2022).

Physical reclamation approaches, such as deep plowing, field leveling, and subsurface drainage, enhance water infiltration and salt leaching. Longitudinal data from Russian rice fields demonstrate that regulating groundwater tables and implementing appropriate irrigation-drainage systems regularly lowers salinity levels (Malysheva et al., 2020). Biological methods such as phytoremediation utilizing halophytes salt-tolerant plants like barley during off-seasons facilitate the biological extraction of salts while enhancing soil organic matter and microbial activity, hence conditioning soils for future rice growth (Singh and Singh, 2022). Moreover, integrated nutrient management, which integrates gypsum application with foliar sprays of urea, zinc sulfate, and potassium sulfate, enhances nutrient availability while minimizing salt effects. Field trials in Bangladesh exhibited a 19% enhancement in rice yield and an improved benefit-cost ratio through the implementation of these strategies (Akhlasur Rahman et al., 2024). These diverse strategies offer an extensive framework for addressing salinity in rice cultivation areas.

## 8 Case studies and success stories

### 8.1 Global perspectives on salinity tolerance

While research on salinity tolerance has progressed considerably, the majority of studies and breeding initiatives have concentrated on areas with particular environmental circumstances or geographical limitations. Consequently, remedies to salt stress have frequently been proposed within restricted geographic boundaries, limiting their relevance to wider, varied areas impacted by soil salinity.

#### 8.1.1 Case study 1: South Asia - the salt-affected plains of India and Pakistan

Soil salinity is a prevalent issue in South Asia, notably in India and Pakistan, particularly in the Indo-Gangetic Plain and coastal regions. Both nations possess extensive areas of saline-sodic soils, where elevated concentrations of sodium chloride (NaCl) and sodium bicarbonate (NaHCO_3_) diminish agricultural output. Approximately 6.73 million hectares of agricultural land in India are impacted by salinity (Sarkar et al., 2024; Singh et al., 2024). The significant variability in soil salinity, coupled with unpredictable monsoons, inadequate irrigation techniques and poor drainage systems, complicates the management of salt stress, leading to the gradual accumulation of salts in the root zone (Verma et al., 2012). Furthermore, indigenous rice and wheat types demonstrate limited resistance to elevated saline, hence constraining yield (Shrivastava and Kumar, 2015). In response, breeding projects in South Asia have concentrated on creating salt-tolerant rice cultivars, exemplified by the ‘FL478’ cultivar, which exhibits tolerance to salinity stress and is being utilized in regions of Pakistan and India (Wu et al., 2024), and the wheat cultivar ‘KRL-19’, which has been effectively cultivated in saline-prone regions (Sheoran et al., 2021). Additionally, advanced irrigation methods, such as the implementation of drip irrigation and improved salinity management techniques, have contributed to alleviating the detrimental impacts of soil salinity (Wang et al., 2023).

#### 8.1.2 Case study 2: Sub-Saharan Africa - the challenges of coastal salinity in Senegal and Egypt

Sub-Saharan Africa, especially coastal areas like Senegal and Egypt, is experiencing rising salinity in soil and water. Saltwater intrusion into the Senegal River Basin has emerged as a critical problem in Senegal, particularly in the lower delta regions (Kabir et al., 2024; Omuto et al., 2024). The implementation of flood irrigation in Egypt, along with increasing sea levels, has led to salt accumulation, threatening essential crops such as rice and wheat (Saqr and Abd-Elmaboud, 2024). These issues are exacerbated by inadequate freshwater supplies and inappropriate irrigation methods, which worsen the salinization of cultivable land. Furthermore, the agricultural industry in numerous African nations is deprived of access to contemporary salinity-tolerant cultivars and sophisticated irrigation technologies that could mitigate salt accumulation in soils (Stein et al., 2023; Ghiardelli et al., 2025; Tarolli et al., 2025). To address these challenges, Senegal has promoted the use of salt-tolerant crops, such as salt-resistant sorghum and millet, to improve agricultural resilience (Guden et al., 2024; Pankaj et al., 2024). In Egypt, investigations into the development of salt-tolerant rice cultivars, including those from the Agricultural Genetic Engineering Research Institute (AGERI), have demonstrated potential in mitigating issues associated with saline water sources (Leawtrakun et al., 2024b). Furthermore, enhanced water management strategies, including subirrigation systems and brackish water treatment technologies, are progressively being implemented to mitigate soil salinization.

#### 8.1.3 Case study 3: the Middle East - salinity management in Iraq and Saudi Arabia

The Middle East suffers significant issues associated with soil salinity, attributable to the region’s extreme temperature, scarce freshwater resources, and heavy dependence on irrigation (Al-Tardeh et al., 2023; Makttoof and Nafawa, 2024). In nations like Iraq and Saudi Arabia, elevated evaporation rates, along with inadequate water quality, result in considerable salinization of both surface and groundwater resources. In Iraq, salinity has made more than 20% of agricultural land unfit for crop cultivation (Benaafi et al., 2024; Makttoof and Nafawa, 2024). The interplay of increasing groundwater salinity and declining irrigation infrastructure in numerous Middle Eastern nations has hindered the maintenance of crop yields (Sameer et al., 2024). Climate change worsens the issues, as elevated temperatures result in heightened evaporation and increased soil salinity (Keutgen, 2023; Hakami-Kermani et al., 2024). To address these challenges, Saudi Arabia has invested in novel solutions, such as utilizing saline groundwater for agricultural purposes. The King Abdullah Initiative for Saudi Agricultural Investment Abroad seeks to research sustainable agricultural methodologies in saline conditions, emphasizing drip irrigation and greenhouse cultivation (Hemdan et al., 2024; Nurbekova et al., 2024; Wang et al., 2024). Similarly, Iraq has implemented salinity management strategies, such as developing salt-tolerant crop varieties, including drought- and salinity-resistant wheat cultivars through conventional breeding and biotechnological approaches (Al-Saadi et al., 2024; Tahir et al., 2024). Moreso, soil reclamation methods, including gypsum application and bio-amendment use, are being tested to alleviate salinity in certain regions (Ali et al., 2024).

#### 8.1.4 Case study 4: Australia - tackling salinity in the Murray-Darling Basin

Australia, especially the Murray-Darling Basin in the southeast, is facing salinity issues due to its dependence on irrigation for agricultural output. Salinity levels in the region have markedly risen over the past few decades, impacting both agricultural productivity and ecosystem health (Walker and Hart, 2024; Nelson et al., 2024). The primary concern in Australia is the extensive salinization of soils resulting from the excessive extraction of groundwater for irrigation purposes (Sanga et al., 2024). This has resulted in elevated salt concentrations in surface water and soils, making extensive regions of farming unproductive (Chanu, 2023). Ineffective land management techniques, such as monocropping and insufficient crop rotation, have further intensified the issue (Hannam, 2024). To address these challenges, Australia has led the advancement of salinity-resistant crop types, including wheat, barley, and cotton, utilizing both traditional breeding and genetic modification techniques (Sharma et al., 2024). Additionally, the Australian government has enacted rules to regulate groundwater extraction and encourage sustainable water management techniques (Abu Romman and Al Kuisi, 2023; Williams et al., 2023). Employing salt-tolerant plants and enhanced irrigation techniques, such as precision agriculture, has alleviated the impacts of salinity in certain areas of the region (Nurbekova et al., 2024).

The case studies from South Asia, Sub-Saharan Africa, the Middle East, and Australia demonstrate the necessity of region-specific techniques for addressing salinity stress in agriculture. Although salt-tolerant varieties and enhanced irrigation techniques have demonstrated efficacy in numerous regions, solutions must be tailored to local conditions, taking into account the distinct problems presented by each region’s soil, water resources, and climate. Along with that, the transfer of information and technology among places impacted by salinity is crucial for formulating global solutions to this escalating problem. With the acceleration of climate change leading to the salinization of agricultural lands globally, it is important to enhance research on salt tolerance, particularly across varied geographic settings, and to execute region-specific solutions that may be adapted and expanded internationally.

### 8.2 Examples of successful deployment of salt-tolerant rice varieties

The introduction of salt-tolerant rice varieties has greatly enhanced agricultural production in saline-affected areas, providing sustainable solutions to food security issues. A prominent instance is the creation of the ‘Ezhome Rice’ varieties ‘Ezhome-1’ and ‘Ezhome-2’ in Kerala, India. These high-yielding, non-lodging organic red rice cultivars were specifically developed for the saline-prone Kaipad farms. Participatory plant breeding with local farmers resulted in these types yielding 3.5 and 3.2 tons per hectare, representing a 60-70% increase compared to standard cultivars (Vanaja et al., 2017). Similarly, the Qingdao Saline-Alkali Tolerant Rice Research and Development Center in China has developed ‘seawater rice’ types that can flourish in salty soils with up to 4 grams of salt per kilogram. By 2021, these types were farmed on 400,000 hectares, yielding an average of 8.8 tons per hectare, thus converting barren saline soils into arable regions (Micu, 2022). The International Rice Research Institute (IRRI) developed salt-tolerant rice strains by hybridizing commercial types with wild species such as *O*. *coarctata*. These hybrids feature specialized leaf glands that excrete excess salt, facilitating agriculture in high-salinity settings and increasing arable land availability (Barona-Edna, 2013). Additionally, the traditional ‘Pokkali’ rice type from Kerala, famous for its exceptional salt resistance, grows to a height of 140 cm and is farmed organically together with prawn culture in coastal areas that experience floods (Wikimedia, 2013).

#### 8.2.1 Regional adoption in South Asia and Sub-Saharan Africa

The use of salt-tolerant rice varieties (STRVs), as listed in **Table 1**, has transformed agriculture in saline-affected areas of South Asia and Sub-Saharan Africa, providing sustainable solutions to food security issues and enhancing agricultural production (Radha et al., 2024; Sellathdurai et al., 2024; Sikder and Khan, 2024; Zayed et al., 2024). In the Mekong River Delta (MRD) of Vietnam, STRVs have significantly minimized the detrimental effects of sea-level rise and salinity intrusion. Advocated by the Consortium for Unfavorable Rice Environments (CURE), these types were implemented in 47% of salinity-affected rice fields during at least one of the two primary growth seasons. Their adoption was significantly elevated in non-irrigated regions and places lacking saline barrier gates, underscoring their effective focus on sensitive ecosystems. Although these varieties reduced production losses under salinity stress, they did not surpass traditional varieties in low-salinity years and frequently garnered lower market prices, indicating a trade-off between stress tolerance and marketability (Paik et al., 2020). In India, the Central Soil Salinity Research Institute (CSSRI) has pioneered the creation of salt-tolerant rice varieties like CSR10 and CSR36, tailored for saline and sodic soils (Kumar et al., 2024; Zayed et al., 2024). These innovations have restored about 1.5 million hectares of salt-affected land, enhancing food grain production by roughly 15 million tons each year (Okur et al., 2023; Farooqi et al., 2024; Nurbekova et al., 2024). CSSRI’s unified strategy merges genetic innovations with smart soil and water management, turning barren lands into thriving agricultural areas and showcasing an integrative approach to tackle salinity (Mishra et al., 2023; Shakar et al., 2024).

**Table 1:** A table listing salt-tolerant rice varieties, their genetic traits, and the regions of adoption.

Salt-Tolerant Rice Variety	Genetic Traits	Regions of Adoption
CSR 36	Contains the Saltol gene (a major gene for salinity tolerance)	India, Bangladesh, Nepal
IR 64	Incorporates QTLs for salt tolerance and high yield under saline conditions	South Asia, Southeast Asia, Africa
NERICA 4	High salinity tolerance, genes for drought and disease resistance	West Africa, Sub-Saharan Africa
Pokkali	Known for high salt tolerance; ability to withstand extreme salinity	India (Kerala), Bangladesh, Sri Lanka
IRRI 123	Incorporates Saltol gene and QTLs for osmotic regulation	Philippines, Southeast Asia
BPT 5204 (Swarna)	Salt tolerance from native genetic traits; improved water-use efficiency	India, Bangladesh, Southeast Asia
Kalarata	Salt tolerance due to salt exclusion mechanisms in roots	Fiji, Pacific Islands
Moroberekan	Known for salt tolerance, with osmotic stress mechanisms	Sierra Leone, Guinea
Swarna Sub1 (Swarna-Submergence Tolerant)	Combination of submergence and salinity tolerance genes	India, Bangladesh, Nepal
Yunnan Bai	High salt tolerance in coastal regions, due to salt exclusion mechanisms	China (Yunnan Province), Southeast Asia
IR 29	Saltol gene and high yield under saline conditions	India, Southeast Asia
Upland Rice (Nerica)	Improved salt tolerance through functional QTLs for salinity and drought	West Africa, Sub-Saharan Africa

The Stress-Tolerant Rice for Africa and South Asia (STRASA) initiative, led by the International Rice Research Institute (IRRI), has enabled the introduction of more than 150 stress-tolerant rice varieties throughout Sub-Saharan Africa. These initiatives have enabled millions of farmers to augment yields and improve resilience to salinity, drought, and flooding. The joint introduction of the salt-tolerant variety ‘KARANADA’ by the Kenya Agricultural and Livestock Research Organization (KALRO) and IRRI has enhanced productivity and income stability in saline-prone regions of Kenya, demonstrating the global efficacy of salt-tolerant rice varieties (STRVs) in mitigating environmental and economic challenges (IRRI, 2023).

## 9 Challenges and future perspectives

### 9.1 Limitations in current breeding and genetic research

Despite advancements in breeding and genetic research, severe impediments in saline-affected regions significantly hinder the deployment of salinity-tolerant rice varieties. These problems must be addressed for productivity and global food security. Rice salt tolerance is polygenic, governed by a complex interplay of genes and quantitative trait loci (QTLs) that influence sodium exclusion, potassium retention, and osmotic adjustment. This complexity hinders the identification, mapping, and integration of beneficial genes into breeding programs, hence impeding progress. The dynamic nature of salinity stress, together with waterlogging, temperature changes, and microclimatic variations, influences the expression of stress-responsive genes, resulting in significant variability in the performance of salt-tolerant rice varieties across different settings and seasons. The dependence on a limited genetic basis, primarily consisting of conventional salt-tolerant donors such as Pokkali, exacerbates these issues by constraining the investigation of innovative tolerance mechanisms and diminishing the adaptability of new types to changing stress conditions.

#### 9.1.1 Limitations of current phenotyping technologies

Phenotyping for salinity tolerance has historically posed a significant challenge in plant breeding, especially for high-throughput screening of extensive populations. Conventional phenotyping techniques, such as manual assessments of plant height, leaf area, and visual evaluations of salt damage, are laborious, time-consuming, and frequently susceptible to subjectivity (Chawade et al., 2019; Gill et al., 2022). Moreover, these approaches fail to comprehensively represent the extensive physiological responses of plants to salinity stress, which encompass complex alterations in ion balance, osmoregulation, and gene expression. A notable limitation is the incapacity of traditional phenotyping methods to deliver real-time, high-resolution data on physiological processes, including transpiration, root development, and ion buildup in reaction to differing salt levels. The magnitude and complexity of contemporary breeding programs require sophisticated and automated phenotyping tools capable of effectively assessing these qualities in both controlled and field environments (Gill et al., 2022). Furthermore, several modern phenotyping methodologies are inadequately equipped to manage the extensive datasets produced by high-throughput screening. This data frequently necessitates comprehensive processing and integration from various sources, potentially creating barriers in breeding processes. Moreover, conventional phenotyping techniques are inadequate for quantifying small differences in salinity tolerance, especially in field situations where environmental variability may hide genotype performance.

#### 9.1.2 Potential of remote sensing and machine learning

Recently, the combined use of remote sensing technologies and machine learning algorithms has surfaced as a potent method to address the constraints of conventional phenotyping. Remote sensing technologies, including drones and satellites, can acquire high-resolution imagery of plant canopies and root systems under salt stress, offering insights on phenotypic characteristics that are challenging to assess manually. Hyperspectral imaging, which captures a wide range of light wavelengths, can identify alterations in leaf pigment composition, water content, and stress-induced biochemical indicators at an early stage (Stutsel et al., 2021; Del Cioppo et al., 2024). This non-invasive method facilitates continuous assessment of plant health over extensive regions, rendering it suitable for field-based phenotyping of salinity tolerance. Furthermore, remote sensing technologies can be integrated with machine learning (ML) models to automate trait extraction and analysis. Training machine learning algorithms on datasets derived from hyperspectral imaging or other remote sensing technologies enables the prediction of essential physiological features associated with salinity tolerance, including leaf area, chlorophyll content, and stomatal conductance. These predictive models may efficiently screen huge populations and find potential genotypes with minimal human interaction (Haq et al., 2023; Mourched et al., 2023). Machine learning enables the amalgamation of diverse data sources, including environmental variables, soil salt levels, and genomic data, to improve the precision of predictions in apropos of plant performance in saline environments. By utilizing features acquired from drone-based imaging data, researchers were able to effectively apply ML algorithms to genotype classification according to salt tolerance (El-Hendawy et al., 2024a; El-Hendawy et al., 2024b). Machine learning can improve the efficiency of salt-tolerant variety production by integrating phenotypic and genotypic data for more informed breeding decisions.

#### 9.1.3 The need for scalable, cost-effective solutions

There are still issues with scalability and cost-effectiveness, but there is great promise for enhancing phenotyping efficiency with remote sensing and machine learning. For smaller research projects or environments with limited resources, the expensive acquisition and maintenance of remote sensing equipment, along with the specialized knowledge needed to operate and analyze the data, can be a significant barrier. Many breeding projects in underdeveloped nations or areas with inadequate technological infrastructure also lack access to advanced phenotyping technologies because these tools are often reserved for well-funded institutions. Addressing these challenges necessitates the development of scalable and cost-effective phenotyping solutions. A promising approach involves the integration of low-cost sensors and imaging systems into current breeding facilities. Small-scale, high-throughput phenotyping platforms utilizing low-cost sensors to measure parameters like leaf temperature, soil moisture, and chlorophyll fluorescence are being developed. These platforms aim to deliver real-time, actionable data at a significantly lower cost compared to more complex remote sensing technologies (Wu et al., 2022; Yassue et al., 2022). These platforms are applicable in both controlled environments and field conditions, providing a more accessible alternative to costly drone or satellite-based systems. In addition, open-source software and cloud-based platforms can play a key role in making phenotyping technologies more accessible. By providing free access to data analysis tools and integrating them with cloud computing infrastructure, it becomes easier for researchers from diverse institutions to collaborate and share phenotypic data. Additionally, community-driven initiatives to develop open-source phenotyping solutions may foster innovation and lower the overall costs associated with technology adoption in breeding programs. To fully harness the potential of these new technologies, there is an urgent requirement for scalable, cost-efficient solutions that can be implemented by breeding programs globally. By addressing these issues, the domain of phenotyping can significantly aid in the advancement of salinity-resistant crops, thereby securing food availability in areas impacted by soil salinization.

### 9.2 Field-level performance and stability

To ensure that salt-tolerant rice varieties exhibit reliable performance in various environments, it is essential that they undergo thorough field-based evaluation. The evaluation of the stability of salinity tolerance traits is most effectively conducted through multi-environment trials (METs), which examine the performance of varieties across diverse climatic and soil conditions. For instance, the Saltol QTL, which has been integrated into widely cultivated varieties such as IR64 and BRRI dhan47, has undergone thorough evaluation in multi-location trials throughout South and Southeast Asia. The trials demonstrated that the Saltol QTL markedly enhances salinity tolerance during the seedling stage; however, its efficacy is influenced by the type of soil and the levels of salinity present (Ho et al., 2016; Yadav et al., 2020). In a similar vein, the FL478 variety, which originates from the salt-tolerant landrace Pokkali, has shown reliable performance in saline-affected areas of India, Bangladesh, and Vietnam, underscoring the significance of field validation. These trials enable researchers to discern varieties that exhibit both salt tolerance and adaptability to various environmental stresses, thereby securing sustained productivity over time (Debsharma et al., 2024; Krishnamurthy et al., 2024). Additionally, field testing can be enhanced through the integration of high-throughput phenotyping technologies, including UAV-based imaging and remote sensing, which facilitate precise monitoring of rice performance in natural field environments. These technologies offer accurate, non-invasive assessments of plant stress responses, enhancing the understanding of salt tolerance mechanisms in practical environments. The integration of METs with modern phenotyping techniques has the potential to connect the insights gained from controlled experiments to the real-world implementation of salt-tolerant rice varieties in the context of agriculture (Del Cioppo et al., 2024; Fu et al., 2024).

### 9.3 Importance of multidisciplinary approaches

Salinity stress in cultivation of rice is complex and varying, requiring a multidisciplinary strategy that integrates genetics, soil science, agronomy, climate modelling, and socioeconomics to provide sustainable solutions. Integrating advancements in genetics, such as the identification of salt-tolerant QTLs and the application of transgenic technologies, with agronomic strategies is essential for achieving success in the field. The implementation of salt-tolerant rice cultivars, such as FL478, alongside efficient irrigation techniques like alternate wetting and drying, significantly improves productivity and resource utilization efficiency. Soil salinity arises from irrigation methods, variations in the water table, and climatic changes, highlighting the necessity for comprehensive soil science strategies. The use of soil additives, such as gypsum and charcoal, alongside breeding programs targeting features like root architecture and ionic exclusion, improves soil fertility and crop resilience.

Predictive climate modelling and stress forecasting improve interventions through the application of remote sensing and GIS technologies. These technologies offer accurate identification of salt-affected regions, guiding breeding and agronomic practices customized to particular geographies. Nevertheless, constrained landholdings and limited access to salt-tolerant seedlings impede technological utilization. Involving politicians, extension agencies, and agricultural communities in participatory breeding efforts fosters equitable transmission of innovation, including farmer preferences and local knowledge to improve uptake and efficacy. Biotechnological innovations and omics technologies have the potential to revolutionize breeding methodologies. However, they must be combined with bioinformatics and high-throughput phenotyping technologies to swiftly uncover context-relevant traits. These solutions synchronize productivity goals with sustainability objectives when incorporated into comprehensive frameworks. Integrating salinity-resistant rice farming with agroforestry in coastal areas improves productivity, increases biodiversity, and strengthens ecosystem resilience. Multidisciplinary approaches create a strong basis for mitigating salt stress and promoting sustainable agriculture and resilient livelihoods in saline-affected areas.

## 10 Conclusion

### 10.1 Summary of key findings

Salinity tolerance in rice presents a complex challenge, necessitating a collaborative strategy that combines genetics, agronomy, and sustainable practices. Significant breakthroughs encompass finding critical QTLs such as Saltol, creating salt-tolerant cultivars such as FL478 and BRRI dhan47, and advancements in gene-editing technologies. Agronomic techniques, such as enhanced irrigation, crop rotation, and organic amendments, augment these genetic remedies, improving soil health and production in saline areas. Case studies from South Asia, Sub-Saharan Africa, the Middle East and Australia, illustrate the effective implementation of salt-tolerant cultivars, showcasing their capacity to convert unproductive regions. Nonetheless, obstacles, including the polygenic characteristics of salt tolerance, restricted genetic diversity, and environmental unpredictability, persist as substantial issues. Future perspectives highlight the necessity for interdisciplinary cooperation, utilizing improved phenotyping, digital agriculture, and participatory breeding to guarantee climate-resilient and sustainable rice-producing systems. These initiatives facilitate the establishment of a more secure and resilient global food system.

### 10.2 Call for global collaboration in addressing salinity challenges

Addressing salinity stress in rice farming requires coordinated global initiatives that combine scientific research, policy formulation, and community involvement. The complexity of salinity tolerance encompassing genetic, physiological, and environmental factors highlights the necessity for cooperative, interdisciplinary approaches. Breeding efforts must integrate advanced genomic techniques with traditional expertise to create hardy varieties suited to various agro-climatic settings. Simultaneously, agronomic advancements, like sustainable irrigation methods, crop diversity, and soil reclamation, must be expanded through inclusive policies and effective extension services. Global research consortia and platforms, including the International Rice Research Institute (IRRI) and Stress-Tolerant Rice for Africa and South Asia (STRASA), have exemplified the transformative potential of collaboration. Expanding these programs will facilitate resource-sharing, capacity building, and equitable access to innovations, especially in vulnerable areas such as South Asia and Sub-Saharan Africa. Policymakers must prioritize financing for climate-smart agriculture and endorse farmer-led participatory initiatives to guarantee the uptake and sustainability of solutions. Furthermore, the use of digital technology, like remote sensing and precision agriculture, in salinity management has the potential to transform the approach to addressing difficulties in the field. This necessitates collaborations across sectors connecting researchers, agribusinesses, and local communities to expedite the shift to resilient rice systems. Global collaboration is essential to confront the escalating issue of salinity. By cultivating partnerships, augmenting knowledge exchange, and investing in scalable innovations, we can ensure sustainable rice production systems and safeguard global food security for future generations.
